# Human Metapneumovirus Genetic Variability, South Africa

**DOI:** 10.3201/eid1107.050500

**Published:** 2005-07

**Authors:** Herbert P. Ludewick, Yacine Abed, Nadia van Niekerk, Guy Boivin, Keith P. Klugman, Shabir A. Madhi

**Affiliations:** * University of the Witwatersrand, Johannesburg, South Africa;; † Laval University, Quebec City, Quebec, Canada;; ‡ Emory University, Atlanta, Georgia, USA

**Keywords:** Keywords: molecular epidemiology, human metapneumovirus, F gene, G gene, Africa, genotype, pneumonia

## Abstract

The molecular epidemiology and genetic diversity of the human metapneumovirus (hMPV) were characterized for a 3-year period (2000–2002) from viruses that were identified in South Africa. Two major genetic groups (A and B) and 2 subgroups (1 and 2) of hMPV were identified, as well as 2–6 possible genotypes within the subgroups. A shift in the predominant group was documented in successive seasons. Whereas the F gene was relatively conserved between subgroups, a high degree of variation was observed in the extracellular domain of the G gene of the virus. The G protein identities between groups A and B were 45.1%–53.1% at the nucleotide level and 22.4%–27.6% at the amino acid level. These results provide evidence for the diversity of both surface glycoproteins of hMPV in Africa, which may be a prerequisite to understanding protective immunity against hMPV.

Human metapneumovirus (hMPV) is an important cause of acute respiratory tract infections worldwide in both children and adults (1–11). It causes annual epidemics during the winter-spring months in temperate regions. Taxonomically, hMPV belongs to the family *Paramyxoviridae*, subfamily *Pneumovirinae*, and is the only known human pathogen of the genus *Metapneumovirus* (1,12). Genetically, its closest relative is the avian pneumovirus type C (1,13,14); however, clinically, it resembles the respiratory syncytial virus (RSV) (15), a common respiratory pathogen classified in the family *Paramyxoviridae*, subfamily *Pneumovirinae*.

Genetic studies on hMPV have demonstrated the presence of 2 distinct hMPV groups and subgroups within these groups (2,4,7,10,14–18); more recently, evidence has been shown that multiple lineages may exist (19,20). Limited available data indicate that both groups can circulate in a single season with the possibility of the predominant group switching in successive seasons (2,4,17,21). Genetic variation in the hMPV attachment glycoprotein (G protein) indicates a high degree of nucleotide variation, which results in amino acid changes (17–19). This sequence variation within the hMPV G gene has been postulated to be due to immunologic pressure. Our study was designed to examine the extent of genetic variation and the circulation pattern of hMPV in a single South African community in 3 consecutive years (2000–2002) by sequence analysis of the 2 surface (F and G) glycoprotein genes from South African strains.

## Materials and Methods

### Specimens

Nasopharyngeal aspirates were obtained from children enrolled in a vaccine efficacy study that was conducted in Soweto, South Africa (22). The samples were obtained from children hospitalized for a lower respiratory tract infection in a 3-year period (2000–2002). Samples were stored at –70°C until processed for this study. Details regarding the cohort of children, procedure for collecting nasopharyngeal aspirate samples, and other viruses isolated from these samples have been published in part (22,23). The samples used in this study were from the entire year and not confined to samples obtained from the winter-spring months. Because of resource constraints, we sequenced a minimum of 30% of the hMPV-positive samples from each month; 92 (45%) of 206 hMPV-positive samples were sequenced for the F gene, and 61 (30%) of 206 were sequenced for the G gene. All samples that were sequenced for the G gene were sequenced for the F gene. Viral RNA was isolated from the stored frozen nasopharyngeal aspirate samples by using the QIAamp viral RNA kit (Qiagen, Inc., Valencia, CA, USA) according to the manufacturer's instructions. The study was approved by the Committee for Research on Human Subjects at the University of the Witwatersrand, South Africa.

### hMPV Detection of F Gene

A nested reverse transcription–polymerase chain reaction (RT-PCR) assay to amplify a fragment of the hMPV F gene was used to detect hMPV. RT-PCR was performed with the SUPERSCRIPT One-Step RT-PCR kit (Invitrogen, Carlsbad, CA, USA) with primers 5´-ATGTCTTGGAAAGTGGTG-3´ (corresponding to nucleotide position 3052–3069 in the NL/1/00 genome accession no. AF371337) and 5´- CCATGTAAATTACGGAGCT-3´ (nucleotide position 3844–3862 in NL/1/00 in genome) under the following conditions: 50°C for 30 min; 94°C for 2 min; 94°C for 30 s, 45°C for 45 s, and 68°C for 1 min for 35 cycles; 68°C for 7 min.

The nested PCR was performed with primers 5´-TCATGTAGCACTATAACT-3´ (nucleotide position 3130–3149) and 5´-TCTTCTTACCATTGCAC-3´ (nucleotide position 3794–3810) under the following conditions: 94°C for 2 min; 94°C, 48°C, and 72°C for 1 min for 30 cycles; and 72° for 7 min. The PCR product was analyzed by electrophoresis on a 2% ethidium bromide–stained agarose gel.

### hMPV Detection of G Gene

The G gene open reading frame (ORF) was amplified with the following primers: HMPVGunivF: 5´-GAGAACATTCGRRCRATAGAYATG-3´ (nucleotide position 6262–6285 of NL/1/00, GenBank accession no. AF371337) and HMPVGunivR: 5´-AGATAGACATTRACAGTGGATTCA-3´ (nucleotide position 7181–7204) under the following conditions: 50°C for 30 min; 95°C for 3 min; 94°C for 1 min, 59°C for 1 min, and 72°C for 2 min for 38 cycles; and 72°C for 7 min. The PCR product was analyzed on a 2% ethidium bromide–stained agarose gel. When necessary to increase the yield for sequencing, a nested PCR was performed with the same primer set.

### Sequencing of hMPV F and G Genes

The PCR product generated for both F and G genes was purified with the QIAquick gel extraction kit (Qiagen, Inc.) and sequenced in both directions by using the nested primers for the F gene and the primers used for detecting the G gene. The PCR product was sequenced by using the BigDye Terminator Cycle sequencing kit (Applied Biosystems, Foster City, CA, USA) on the ABI 310 Genetic Analyzer (Applied Biosystems).

### Phylogenetic Analysis

Nucleotide sequence alignments were generated with the ClustalX 1.81 software (24). Phylogenetic analysis was performed by using MEGA version (2.1) (25). Strains from the Netherlands, NL/1/00, NL/17/00, NL/1/99, and NL/1/94 (GenBank accession nos. AF371337, AY304360/AY296021, AY304361/AY296034, and AY304362/AY296060, respectively) and Canada, CAN97-83, hMPV13-00, CAN98-75, and hMPV33-01 (GenBank accession nos. AY485253/AY145296, AY485232, AY485245/AY145289, and AY485242, respectively) were used as prototypes of the 2 groups and subgroups. The hMPV sequences for the F and G protein genes presented in this article have been deposited in GenBank under the accession numbers AY694693–AY694784 and AY848859–AY848919, respectively.

## Results

A total of 2,802 samples collected in the 3-year study period were analyzed by RT-PCR for hMPV by amplification of the F gene protein. hMPV was identified in 206 (7.4%) samples in the 3-year period. One hundred one (9.6%) of the 1,057 samples from the year 2000, 82 (7.3%) of 1,128 samples from 2001, and 23 (3.7%) of 617 samples from year 2002 were positive for hMPV.

### Molecular Epidemiology of hMPV

We examined the circulation pattern of hMPV during the 3 years by sequencing part of the hMPV F gene and performing phylogenetic analysis for 92 hMPV-positive samples. These samples, numbering 40, 34, and 18 from years 2000, 2001, and 2002 respectively, were distributed over all the months when hMPV was identified and accounted for ≈30%–100% of the samples from each month. Phylogenetic analysis indicated 2 major groups (A and B), each divided into 2 subgroups (1 and 2), causing a complex circulation pattern over the course of the study. Both groups A and B viruses cocirculated throughout the study period. Of the 92 hMPV-positive samples that were categorized, 56 (60.9%) of the viruses belonged to group A and 36 (39.1%) to group B.

During the 2000 epidemic, subgroups B2 and A2 cocirculated, with 72.5% of the circulating viruses belonging to subgroup B2. In 2001 subgroups A1, A2, and B2 cocirculated. Subgroup B2 virus significantly declined (4 [11.8%] of 34) in 2001 compared to 2000 (29 [72.5%] of 40), p<0.0001. Subgroup A1 emerged as the dominant strain, causing 67.7% of infections, compared to 20.5% observed for subgroup A2 and 11.8% for subgroup B2. Subgroup A1 also dominated in 2002, causing 83.3% of infections, and cocirculated with the emergent subgroup B1.

Sixty-one (66.3%) of the 92 hMPV-positive samples sequenced for the F gene were further sequenced for the G gene (Figure). Phylogenetic analysis of both the F and G gene nucleotide sequences also showed 2 major genetic groups (A and B) that could be further divided into 2 subgroups (1 and 2). The existence of these 2 major genetic groups was strongly supported by bootstrap values (100% of bootstrap replicas into 2 major groups and 99%–100% of bootstrap replicas into 2 minor subgroups).

From the topology of the trees, subgroup B2 was the most divergent. Although the South African hMPV clustered with both Canadian and Netherlands prototypes, the South African subgroup A1 virus clustered more closely with the Canadian prototype.

From the topology of the tree and supported by strong bootstrap values (70%–100%), the subgroups can be further divided into genotypes. We attempted to group the G sequences of the 4 subgroups into genotypes by using the criteria previously described for RSV (26), in which sequences were arbitrarily considered a genotype if they clustered together with bootstrap values of 70% to 100% (internal nodes at the internal branches). When these criteria were used (only on South African isolates), subgroup A1 could be divided into 5 genotypes, subgroup A2 into 2 genotypes, B1 into 2 genotypes, and B2 into 6 possible genotypes.

### Genetic Variation in South African hMPV Isolates

The estimated nucleotide and amino acid identities showed a high percentage of identity for the F gene and more variability for the G gene (Table). The estimated identities for the F gene between the 2 major groups, A and B, were 83%–85% at the nucleotide level and 93.2%–95.8% at the amino acid level. In contrast, the G gene estimated identities were 45.1%–53.1% at the nucleotide level and 22.4%–27.6% at the amino acid level. There was also a higher percentage of identity between members of the same group (e.g., A1–A2) for the F gene than for the G gene (Table).

**Table:** Human metapneumovirus F and G gene nucleotide and amino acid identities of South African strains in 3 consecutive years (2000–2002)

Gene	Subgroups	% nucleotide (amino acid) identities			
		A1	A2	B1	B2
F	A1	99–100 (98.4–100)	93–95 (96.3–97.9)	83.8–84.1 (93.2–94.3)	82.7–84.5 (94.3–95.8)
	A2		99–100 (99.4–100)	83–83.8 (94.3)	83.1–85 (95.3–95.8)
	B1			98–100 (100)	93–95 (98.4)
	B2				96–100 (99.4)
G	A1	95.4–100 (87.5–100)	72.8–74.7 (55–63.6)	45.9–47.8 (24.3–26.2)	47.4–48.7 (22.4–26.7)
	A2		95–100 (88.6–98.1)	50.3–51.8 (25.4–27.7)	51–53.1 (23.6–27.6)
	B1			93.2 (87.9)	77.4–80.5 (58.2–62.8)
	B2				93.2–100 (82.8–100)

Amino acid alignments of hMPV F gene were compared to those of prototype isolates from the Netherlands and Canada (data not shown). Cysteine residues were conserved in all South African strains at positions 60 and 182. Group-specific amino acid residue at positions 122, 135, 139, 167, 175, and 233 differentiated between groups A and B. Further amino acid substitutions at various positions were exclusive to subgroups A1 (amino acids [aa] 61, 82, 143), A2 (aa 61, 143, 185), and subgroups B1 (aa 46, 143, 179) and B2 (aa 143).

**Figure A1:**
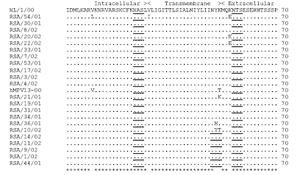
Alignment of the G proteins of representative samples of South African human metapneumovirus (hMPV) isolates and prototypes sequences from the Netherlands (NL/1/00, NL/1/99, NL/1/94, and NL/17/00) and Canada (hMPV13-00, CAN97-83, hMPV33-01, and CAN75-98). Only amino acid residues that differed from the Netherlands' prototypes for each subgroup are shown. Identical amino acids are represented by periods; dashes indicate gaps. Arrows above the alignment indicate the proposed intracellular, transmembrane and extracellular domains. Potential N-linked glycosylation sites are underlined. Numbers indicate the amino acid position in the G open reading frames corresponding to the Netherlands' prototype isolates. Download PDF [46 KB, 5 pages].

The predicted G ORF amino acid alignments of selected South African strains with prototypes from the Netherlands and Canada are shown in Figure A1. Sequence variation due to nucleotide substitutions and insertions led to variable lengths in polypeptides, which ranged from 228 aa residues (subgroup A2) to 240 aa residues (subgroup B2). The hMPV G ORFs of subgroups A2 and B1 terminated at the TAA codon, whereas the subgroup B2 isolates terminated at the TAG codon. For both genetic groups A and B, a cysteine residue was present in the intracellular domain. In addition, group B isolates had a cysteine residue in the extracellular domain except in 2 isolates (RSA/71/00 and RSA/90/00).

The region of the predicted G ORF sequenced in this study had a high serine and threonine content (30.7%–34.9% for group A, 30.6%–36.6% for group B isolates). Proline content varied among the subgroups: 7.6%–9.0% for subgroup A2, 9.0%–9.9% for subgroup A1, 7.8%–8.7%for subgroup B1, and 3.7%–5.2%, the lowest content, for subgroup B2. Only 1 potential N-linked glycosylation site was conserved at the junction of the intracellular and transmembrane domains.

## Discussion

Genetic variability is a strong indicator of positive selection and affects the ability of a virus to continue circulating in a population. This variability poses a challenge for future vaccine development that relies on worldwide molecular epidemiologic studies. Recently, the hMPV G gene was shown to be highly variable, particularly in the extracellular domain, as a result of nucleotide substitutions, insertions, and the use of alternative termination transcription codons (17–19). Limited data have also indicated that the 2 groups of hMPV cocirculate and that different subgroups may predominate from year to year (2,4,17,21).

We report on the largest community-based phylogenetic study of hMPV for both surface glycoproteins and provide evidence on the circulation pattern of hMPV in a single African community in 3 consecutive seasons. We also provide evidence for the presence of multiple lineages and genotypes of hMPV, as has been previously observed for other respiratory viruses such as RSV (26,27).

Phylogenetic analysis based on nucleotide sequences of the F and G ORFs of the South African strains demonstrated the existence of 2 groups (A and B) and 2 subgroups (1 and 2). Using the criteria described for the existence of multiple lineages for RSV (26), we demonstrated that multiple lineages of hMPV are circulating in South Africa; however, these lineages need to be characterized at the antigenic level and the clinical impact characterized.

Strains from both hMPV groups cocirculated in South Africa (Soweto, Johannesburg), but not all 4 subgroup viruses cocirculated in a single year, evidence for a complex circulation pattern that permits hMPV to evade preexisting immunity. In 2000, subgroups A2 and B2 cocirculated; in 2001, A1, A2, and B2 cocirculated; and in 2002, A1 and B1 cocirculated. A switch in predominant subgroup from B2 to A1 was observed from the 2000 to the 2001 epidemic. Subgroups A2 and B2 also declined in subsequent years, and subgroup B1 emerged in 2002. The absence of subgroup B1 in previous years may have been due to preexisting community immunity rather than diagnostic assay limitations, as has been speculated (21). Noting the trend in our results, we speculate that the emergence of subgroup B1 virus may eventually have led to the displacement of subgroup A1 as the dominant viral strain in subsequent years. Similar findings in changes of the dominant group of virus that emerges, fostered by a high prevalence of preexisting community immunity to the other major viral group, have been documented for RSV (26–29). Our study, and another from the Southern Hemisphere (21) showed a high prevalence of subgroup A1 in 2001, a finding that suggests that specific strains may coexist across geographic areas in a given epidemic.

hMPV in this study was sequenced directly from specimens, which avoided any amino acid changes due to cell culture adaptation of the viral surface proteins. Although we only sequenced part of the F gene ORF, our results concur with those of a previous study that sequenced the full-length F gene and showed it to be highly conserved (18). In contrast, a high degree of variation was observed for the G gene at the nucleotide and amino acid levels. The sequence variation in the G gene was due to nucleotide substitutions, in-frame insertions, and the use of alternative termination transcription codons. The in-frame insertions we observed suggest that the nucleotide changes previously seen (17) were not due to the passage of hMPV in cell culture. Structural features of the G protein for both groups of South African strains were similar to those observed by others (17,19) with a high serine-threonine content (31%–36%) and variable numbers and positions of N-linked glycosylation sites. The N-linked glycosylation site at the junction between the intracellular and transmembrane domains (position 30–32) was the only conserved site among all groups. We also only observed 1 conserved cysteine residue in the intracellular domain, at amino acid position 27 of the G gene. The second cysteine residue in the extracellular domain, at position 65, previously reported to be present in all group B isolates (17), was absent from 2 South African group B isolates. Hydrophobicity plot data (data not shown) were also similar for both groups A and B, and as reported by others (17,19).

Although our study is limited by the number of hMPV strains sequenced in the study period and we performed partial sequencing of the F gene, we showed that the circulation pattern of hMPV is complex and that the circulation of multiple lineages may suggest an attempt at evasion of preexisting immunity. Our findings also suggest that extended surveillance, over many years, may be necessary to understand the molecular epidemiology of hMPV in any given geographic area.

## Supplementary Material

Figure A1 Appendix Figure in PDF format.

## Figures and Tables

**Figure:**
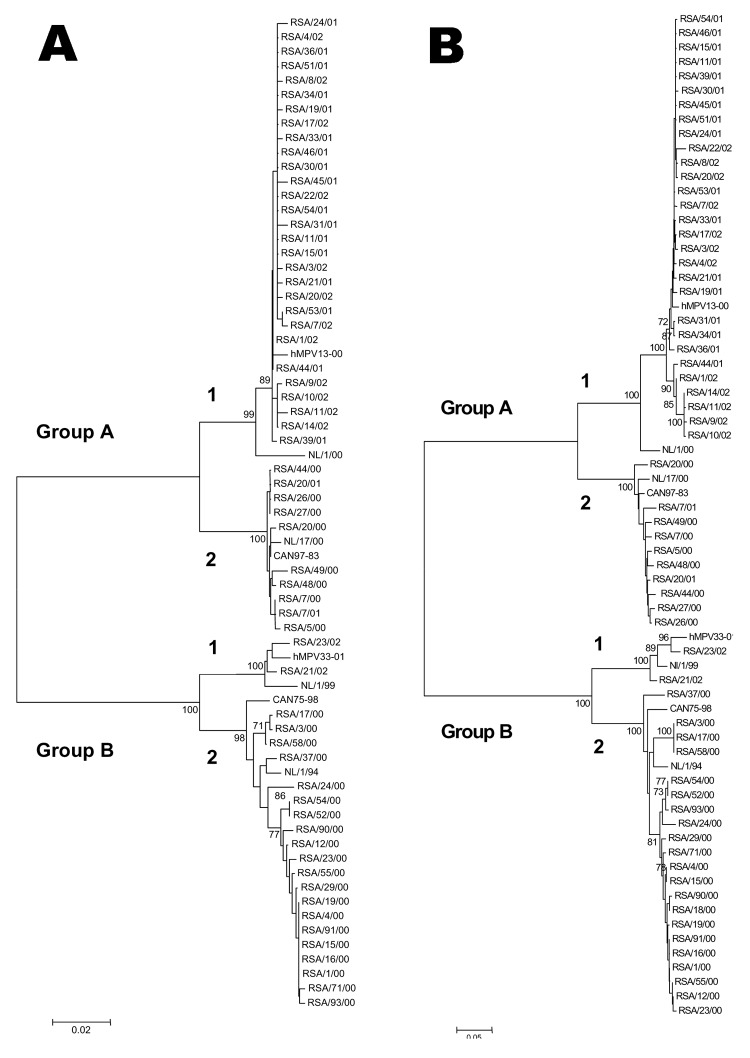
Neighbor-joining trees based on nucleotide sequences from A) the partial F gene and B) the G gene open reading frame from 61 South African human metapneumovirus (hMPV) isolates. The trees were computed with MEGA version 2.1 with the nucleotide Kimura 2-parameters. Bootstrap probabilities for 500 replicas are shown at the branch nodes. Only values of 70% to 100% are indicated. Isolates from South Africa are indicated by RSA, followed by the isolate number and year (e.g., RSA/18/02). The viruses from Canada (CAN97-83, hMPV13-00, CAN75-98, and hMPV33-01) and the Netherlands (NL/1/00, NL/17/00, NL/1/99, and NL/1/94) are prototypes from each subgroup.
