# Supplementary material for: Human Metapneumovirus Genetic Variability, South Africa
Source: Emerg Infect Dis. 2005 Jul;11(7):1074–8. doi: 10.3201/eid1107.050500 (PMC3371776; doi:10.3201/eid1107.050500)
Supplement: Figure A1 — Appendix Figure in PDF format. [file 05-0050-FA1.pdf]

Subgroup A1

```
                                 Intracellular ><    Transmembrane  >< Extracellular
NL/1/00          IDMLKARVKNRVARSKCFKNASLVLIGITTLSIALNIYLIINYKMQKNTSESEHHTSSSP 70
RSA/54/01        .......L...............I......................E............. 70
RSA/30/01        ............................................................ 70
RSA/8/02         ............................................................ 70
RSA/20/02        ..............................................K............. 70
RSA/22/02        ..............................................E............. 70
RSA/33/01        ............................................................ 70
RSA/7/02         ............................................................ 70
RSA/53/01        ............................................................ 70
RSA/17/02        ............................................................ 70
RSA/3/02         ............................................................ 70
RSA/4/02         ............................................................ 70
hMPV13-00        .......V...................................T................ 70
RSA/21/01        ...........................................K................ 70
RSA/19/01        ............................................................ 70
RSA/31/01        ............................................................ 70
RSA/34/01        ............................................................ 70
RSA/36/01        ..........................................H................. 70
RSA/10/02        ..........................................YT................ 70
RSA/14/02        ............................................................ 70
RSA/11/02        ............................................................ 70
RSA/9/02         ............................................................ 70
RSA/1/02         ............................................................ 70
RSA/44/01        ............................................................ 70
                 ******* *************** ******************  ** *************

NL/1/00          MESSRETPTVPTDNSDTNSSPQHPTQQSTEGSTLYFAASASSPETEPTSTPDTTNRPPFV 130
RSA/54/01        ..................PGS.Y...........H..................AS..L.. 130
RSA/30/01        ............................................................ 130
RSA/8/02         ..................................................L......... 130
RSA/20/02        ..................................................P......... 130
RSA/22/02        .............................G.....P........................ 130
RSA/33/01        .............................E.....F.................T...... 130
RSA/7/02         ............................................................ 130
RSA/53/01        ............................................................ 130
RSA/17/02        ...............................F............................ 130
RSA/3/02         ............................................................ 130
RSA/4/02         ...............................S............................ 130
hMPV13-00        .........................................................P.. 130
RSA/21/01        ....................................V....................L.. 130
RSA/19/01        ....................................A....................... 130
RSA/31/01        ......................H..................................... 130
RSA/34/01        ...............................................I............ 130
RSA/36/01        ..........L...........Y........................T............ 130
RSA/10/02        ..........PM..........H............S...T...G......S......PL. 130
RSA/14/02        ............................................................ 130
RSA/11/02        ............................................................ 130
RSA/9/02         .........................................................A.. 130
RSA/1/02         ......................Y............F.......E.............PF. 130
RSA/44/01        .......................................A.................... 130
                 **********  ******   * ****** * **   ** *** *** ** **  **  *

NL/1/00          DTHTTPPSASRTKTSPAVHTKNNPRTSSRTHSPPRATTRTARRTTTLRTSSTRKRPSTAS 190
RSA/54/01        ............R..........L.I.P......W.M...V.G........I.....A.. 190
RSA/30/01        ............................................................ 190
RSA/8/02         ...................................................T........ 190
RSA/20/02        ............................................................ 190
RSA/22/02        .......................P...............................L.... 190
RSA/33/01        .......................L...........................I...P.... 190
RSA/7/02         ............................................................ 190
RSA/53/01        ............................................................ 190
RSA/17/02        ............................................................ 190
RSA/3/02         ...................................................T........ 190
RSA/4/02         .......................P...........................I........ 190
hMPV13-00        .......................LK................................T.. 190
RSA/21/01        ........................R................................... 190
RSA/19/01        ..................Y....P....K............................... 190
RSA/31/01        ..................H....L.T..R..................H...T........ 190
RSA/34/01        ...............................................R............ 190
RSA/36/01        .......................P.................................... 190
RSA/10/02        .........................I......S...T....................... 190
RSA/14/02        ................................P........................... 190
RSA/11/02        ............................................................ 190
RSA/9/02         ............................................................ 190
RSA/1/02         ...................................................I........ 190
```

```
RSA/44/01         ............K............T.................................. 190
                  ************ ***** ****   *  *** * * *** * **** *** *** * **

NL/1/00           VQPDISATTHKNEEASPASPQTSASTTRIQRKSVEANTSTTYNQT 235
RSA/54/01         ....S......H....SV........A.P...------------- 222
RSA/30/01         ................P...............------------- 222
RSA/8/02          ................................------------- 222
RSA/20/02         ................................------------- 222
RSA/22/02         ................................------------- 222
RSA/33/01         ................................------------- 222
RSA/7/02          .......................-N.......------------- 221
RSA/53/01         .......................AS.......------------- 222
RSA/17/02         ................................------------- 222
RSA/3/02          ................................------------- 222
RSA/4/02          ................................------------- 222
hMPV13-00         ......T.........................GMEASTSTTHNQT 235
RSA/21/01         .R....A.........................------------- 222
RSA/19/01         .Q..............................------------- 222
RSA/31/01         ................................------------- 222
RSA/34/01         ................................------------- 222
RSA/36/01         ......................P.........------------- 222
RSA/10/02         ......................S.......GR------------- 222
RSA/14/02         ................................------------- 222
RSA/11/02         ...........Q....................------------- 222
RSA/9/02          ...........H....................------------- 222
RSA/1/02          ................................------------- 222
RSA/44/01         ................................------------- 222
                  * ** * **** ****  ****   * * *

Subgroup A2
                Intracellular ><    Transmembrane  >< Extracellular
NL/17/00          VKNRVARSKCFKNASLILIGITTLSIALNIYLIINYTIQKTTSESEHHTSSPPTEPNKEA 77
RSA/7/00          .......................................................S.... 77
RSA/26/00         .........................M.................................. 77
RSA/27/00         .........................I.................................. 77
RSA/7/01          .......................................................P.... 77
RSA/20/01         .......................................................S.... 77
RSA/44/00         ............................................................ 77
RSA/5/00          ............................................................ 77
RSA/48/00         ............................................................ 77
RSA/49/00         ............................................................ 77
CAN97-83          .........................................S.................. 77
RSA/20/00         .....................................M...T.................. 77
                  ************************* *********** *** ************* ****

NL/17/00          STISTDNPDINPSSQHPTQQSTENPTLNPAASASPSETEPASTPDTTNRLSSVDRSTAQP 137
RSA/7/00          ............N...S........................................... 137
RSA/26/00         ............................................................ 137
RSA/27/00         .....N...................................................... 137
RSA/7/01          .....................................I..............A....... 137
RSA/20/01         .....................................T..............V....... 137
RSA/44/00         ......................................................S..... 137
RSA/5/00          ....................Y.................................R..... 137
RSA/48/00         ....................S.........................I........P.... 137
RSA/49/00         ..............................................T........S.... 137
CAN97-83          ................P...............V........................... 137
RSA/20/00         ................................A...................A....... 137
                  ***** ****** *** *** *********** **** ******** ***** *  ****

NL/17/00          SESRTKTKPTVHTINNPNTASSTQSPPRTTTKAIRRATTFRMSSTGKRPTTTLVQSDSST 197
RSA/7/00          ..........A..R...S....I.....A..........L.................... 197
RSA/26/00         .......E..V...........T.L................................... 197
RSA/27/00         .......................................F.................... 197
RSA/7/01          ..............................M.....................P......I 197
RSA/20/01         ........................F.....T.....................S......T 197
RSA/44/00         ....A..............T.....S..........................L....... 197
RSA/5/00          ....T..K........S..S.....P..........................S....... 197
RSA/48/00         ...................A.....Q..........................L....... 197
RSA/49/00         ........................SP.................................. 197
CAN97-83          ................P...................................S....... 197
RSA/20/00         ........L..............................L.T..........P....I.. 197
                  **** **  * ** **  * ** *  ** * ******** * ********** **** *
```

```
NL/17/00        TTQNHEETGSANPQASASTMQN--------- 219
RSA/7/00        ................V.....QHTNNTKPN 211
RSA/26/00       ......................QHTNNTKPN 211
RSA/27/00       ......................QHTNNTKPN 211
RSA/7/01        ................A.....QHTNNTKPN 211
RSA/20/01       ..........T.....V.....QHTNNTKPN 211
RSA/44/00       ..........A...........QHTNNIKPS 211
RSA/5/00        ................A.....QHTNNIKPN 211
RSA/48/00       ......................QHTNNIKPN 211
RSA/49/00       ...................I..QHTNNIKPN 211
CAN97-83        ................V..M..HTNN-IKPN 210
RSA/20/00       ................A.....--------- 202
                ********** ***** ** **
```

```
Subgroup B1
               Intracellular ><    Transmembrane   >< Extracellular
NL/1/99         SRCYRNATLILIGLTALSMALNIFLIIDHATLRNMIKTENCANMPSAEPSKKTPMTSTAG 84
hMPV33-01       ............................................................ 84
RSA/23/02       ............................................................ 84
RSA/21/02       .............................................P.........I.... 84
                ********************************************* ********* ****

NL/1/99         PNTKPNPQQATQWTTENSTSPVATPEGHPYTGTTQTSDTTAPQQTTDKHTAPLKSTNEQI 144
hMPV33-01       .S...................A..L......E....P...........Y..LS....... 144
RSA/23/02       ...............................G................H...P....... 144
RSA/21/02       .N......................P....H.............................. 144
                * ******************* ** **** * **** *********** **  *******

NL/1/99         TQTTTEKKTIRATTQKREKGKENTNQTTSTAATQTTNTTNQIRNASETITTSDRPRTDTT 204
hMPV33-01       .......K.T...P.R.K.......Q..............................I.I. 204
RSA/23/02       .......R.....TPKKG.R.....P..............................T.T. 204
RSA/21/02       .......K......QRRE.......Q................K..I............S. 204
                ******* * ***     * ***** **************** ** ********** * *

NL/1/99         TQSSEQTTRATDPSSPPHHA------- 224
hMPV33-01       ...........E.GF.....RRGAGPR 231
RSA/23/02       ..........A...SS.Y..RRGAGPR 231
RSA/21/02       ..........TD.S.P.HR.QGSAKPK 231
                **********  *   *  *
```

Subgroup B2

```
                 Intracellular ><      Transmembrane  >< Extracellular
NL/1/94         SSKCYRNATLILIGLTALSMALNIFLIIDYAMLKNMTKVEHCVNMPPVEPSKKTPMTSAV 83
CAN75-98        ..............S................TS........................... 83
RSA/4/00        ..............L.................L..V........................ 83
RSA/29/00       ............................................................ 83
RSA/90/00       .........................................W.................. 83
RSA/71/00       ............................................................ 83
RSA/12/00       .........................................C............L..... 83
RSA/23/00       ............................................................ 83
RSA/54/00       ...................................M..................P..... 83
RSA/93/00       ............................................................ 83
RSA/24/00       ............................................................ 83
RSA/3/00        ...............................M..........I................. 83
RSA/17/00       ............................................................ 83
RSA/37/00       ..........................................V................. 83
                ************** ****************  ** *****  *********** *****

NL/1/94         DLNTKPNPQQATQLAAEDSTSLAATSEDHLHTGTTPTPDATVSQQTTDEYTTLLRSTNRQ 143
CAN75-98        .P............TT.........L.......................H......T... 143
RSA/4/00        .L...L...................S.N.......Q.S...I..............I... 143
RSA/29/00       ............................................................ 143
RSA/90/00       ....................................I....................... 143
RSA/71/00       ............................................................ 143
RSA/12/00       ................................................K........... 143
RSA/23/00       ................................................E........... 143
RSA/54/00       .......L...........................P........................ 143
RSA/93/00       .I............N............................................. 143
RSA/24/00       YL.....P......T............D................................ 143
RSA/3/00        D.............................L.....IP...V...A..........T... 143
RSA/17/00       ........................................T................... 143
RSA/37/00       .S...........................S......TS.T.....T.............. 143
                  *** * ******  ** **  ** * *  ****   * * *** **  ****** ***

NL/1/94         TTQTTTEKKPTGATTKKETT-----RTTSTAATQTLNTTNQTSYVREATTTSARSRNSAT 198
CAN75-98        .....A.....R....KETT-----..........L.......NGR...........N.. 198
RSA/4/00        .....T.....G.I..KEKE---TT..........P.........K.......G...G.. 200
RSA/29/00       ................KEKE---TT............................R...... 200
RSA/90/00       ................KEKE---TT.........................I......... 200
RSA/71/00       I...............KEKE---TT.........................T......... 200
RSA/12/00       T...............KEKE---TT................................... 200
RSA/23/00       ................KEKE---TT................................... 200
RSA/54/00       ................KEKE---TT....................R.............. 200
RSA/93/00       ................KEKEKETTT................................... 203
RSA/24/00       ................KEKE---TT....I.........................K.... 200
RSA/3/00        ................-EKE---TT....T.....L...................R..V. 199
RSA/17/00       ................KEKE---TT................................... 200
RSA/37/00       .......N......A.---E---TT..........P..............K...P..GA. 197
                 **** * *** *  *         **** ***** *******   **** **   *  *

NL/1/94         .QSSDQTTQAADPSSQPHHTQKSTTTTYNTDTSSPSS 235
CAN75-98        ..S.............SQ.........H......... 235
RSA/4/00        ..N....I.......KPH................... 237
RSA/29/00       ..................................... 237
RSA/90/00       ..................................... 237
RSA/71/00       ..................................... 237
RSA/12/00       ..................................... 237
RSA/23/00       ...........................Y......... 237
RSA/54/00       ............S..............H......L.. 237
RSA/93/00       ............P........................ 237
RSA/24/00       .......T...................Y......P.. 237
RSA/3/00        .........T.....Q.Y................SN. 236
RSA/17/00       ..................................... 237
RSA/37/00       .R..........Q......................S. 234
                *  **** * ** **   ********* ******  *
```
